# Colorization-Based RGB-White Color Interpolation using Color Filter Array with Randomly Sampled Pattern

**DOI:** 10.3390/s17071523

**Published:** 2017-06-28

**Authors:** Paul Oh, Sukho Lee, Moon Gi Kang

**Affiliations:** 1 Department of Electrical and Electronic Engineering, Yonsei University, 50 Yonsei-ro, Seodaemun-Ku, Seoul 03722, Korea; phoenix820@naver.com; 2 Department of Software Engineering, Dongseo University, 47 Jurye-ro, Sasang-Ku, Busan 47011, Korea; petrasuk@gmail.com

**Keywords:** RGB-White, color interpolation, colorization, low light conditions, randomly sampled pattern, color filter array

## Abstract

Recently, several RGB-White (RGBW) color filter arrays (CFAs) have been proposed, which have extra white (W) pixels in the filter array that are highly sensitive. Due to the high sensitivity, the W pixels have better SNR (Signal to Noise Ratio) characteristics than other color pixels in the filter array, especially, in low light conditions. However, most of the RGBW CFAs are designed so that the acquired RGBW pattern image can be converted into the conventional Bayer pattern image, which is then again converted into the final color image by using conventional demosaicing methods, i.e., color interpolation techniques. In this paper, we propose a new RGBW color filter array based on a totally different color interpolation technique, the colorization algorithm. The colorization algorithm was initially proposed for colorizing a gray image into a color image using a small number of color seeds. Here, we adopt this algorithm as a color interpolation technique, so that the RGBW color filter array can be designed with a very large number of W pixels to make the most of the highly sensitive characteristics of the W channel. The resulting RGBW color filter array has a pattern with a large proportion of W pixels, while the small-numbered RGB pixels are randomly distributed over the array. The colorization algorithm makes it possible to reconstruct the colors from such a small number of RGB values. Due to the large proportion of W pixels, the reconstructed color image has a high SNR value, especially higher than those of conventional CFAs in low light condition. Experimental results show that many important information which are not perceived in color images reconstructed with conventional CFAs are perceived in the images reconstructed with the proposed method.

## 1. Introduction

Up to the present, most digital imaging systems obtain a full color image using a single sensor to reduce the cost and size of the system. The surface of the sensor is covered by a patterned color filter, called the color filter array (CFA), where each pixel in the pattern passes through only a certain color corresponding to a particular spectral band. The most widely used CFA pattern is the Bayer CFA pattern [1], where each pixel passes through only one of the primary colors (Red, Green and Blue), and the ratio of the numbers of the R, G, and B pixels is 1:2:1. Since each pixel captures only one of the primary colors, the other missing colors have to be obtained by interpolating neighboring colors. A lot of research has been done to find a good interpolation technique that reconstructs the missing colors as good as possible with respect to the given color filter array [2,3,4,5,6,7,8,9,10,11].

In order to use spectral information other than the RGB channel spectrum, a lot of study have been done on multispectral filter arrays [12,13,14,15,16]. By utilizing the additional spectral information obtained by the multispectral filter array, it is possible to reduce color artifacts [12,13], discriminate objects more precisely [14], and obtain invisible information such as that of NIR [15,16]. Simultaneously with these researches, demosaicing methods for various patterns have been also developed [17,18,19,20,21].

**Figure 1:**
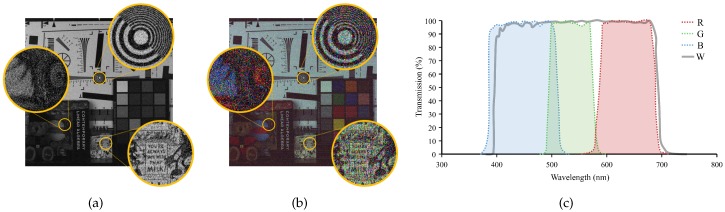
(**a**) W channel and (**b**) RGB channel images in the low light condition(1lux); and (**c**) the transmission graph of the R, G, B, and W color filters in the visible spectrum band.

Recently, new CFA patterns [22,23,24,25] and demosaicing methods [18,19,20,21] have been proposed which also contain panchromatic “white” (W) pixels in the pattern. The W pixel has a much wider spectral band than the R, G, and B pixels as shown in Figure 1, and therefore can absorb more photons than other color pixels, making it more robust against the image noise. There are many causes of image noise, which is produced by the sensors and circuitry of a digital imager [26,27]. The fact that the W pixels are more robust against the noise is due to the fact that the signal to noise ratio (SNR) increases as the number of captured photons increases. This is especially true in low light condition, since in low light condition, the energies of the signal and the noise are similar and increasing the energy of the signal has a great effect in the SNR value. With conventional methods, the conversion from the acquired RGBW pattern to the color image is usually done in two steps: first, the conversion of the RGBW pattern to the widely used Bayer CFA pattern, and second, the conversion of the Bayer CFA pattern to the color image. The reason that the RGBW pattern is first converted to the Bayer CFA pattern is that this conversion is relatively easy and that numerous demosaicing and denoising algorithms [2,3,4,5,6,7,8,9,10,11,28,29] exist for the Bayer CFA pattern. Furthermore, conventional Bayer-oriented imaging signal processors(ISP) can be utilized with this approach. However, the reconstructed color image is degraded by this two-step conversion, since both steps introduce aliasing artifact and color distortion, which aggravates when combined together.

In this paper, we propose a new RGBW CFA pattern that has a large ratio of W pixels in the pattern and also propose the corresponding demosaicing method for this pattern. Due to the large ratio of W pixels in the CFA pattern, a large amount of light can be absorbed by the CFA pattern, resulting in a reconstructed color image with a high SNR value. Therefore, the proposed RGBW CFA pattern shows a great advantage over conventional ones in low light condition, since in low light condition, the quality of the reconstructed color image depends critically on the energy of the light absorbed by the CFA. The proposed demosaicing method for the proposed CFA pattern is based on the colorization technique which was initially proposed for automatic colorization of a gray image by the computer graphic society. The proposed demosaicing method is capable of recovering the RGB color channels from a small set of color pixels, which is the main reason that the proposed CFA pattern can have a relatively small set of RGB pixels while leaving a large space left for W pixels. Furthermore, the demosaicing method directly converts the proposed RGBW CFA pattern image to the color image, and therefore, the aliasing artifact introduced by the two step conversion in conventional RGBW CFA methods is reduced.

## 2. Preliminaries

To understand the proposed method, the following preliminaries have to be understood.

### 2.1. RGB-White CFA

**Figure 2:**
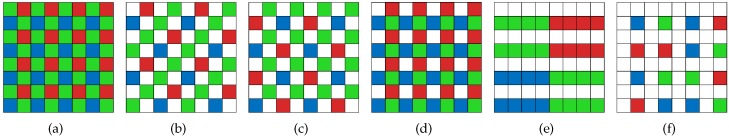
Various CFA patterns: (**a**) Bayer [1]; (**b**) Sony RGBW [22]; (**c**) Yamagami [23]; (**d**) Gindele [24]; (**e**) Compton [25]; and (**f**) Proposed RGBW.

A conventional Bayer CFA pattern contains twice as many G pixels as the R and B pixels as shown in Figure 2a. This is due to the fact that the spectral band of the G channel lies between the R and B channels, and therefore, has a relatively high spectral correlation with those channels. Furthermore, the G channel is highly related with the luminance channel of the image, and therefore, has a large influence on the perceptual quality of the color image, thus, it is advantageous that the G channel possesses a large portion of the CFA pattern.

However, as can be seen in Figure 1, compared to the G channel, the W channel has a much wider spectral band and also a larger overlapping region with the R and B spectral bands. Due to the wide spectral band, the W channel absorbs more light than other channels, and therefore, has a larger SNR value than other channels, which is especially true in low light condition. Furthermore, the W channel can be regarded as the luminance channel, and contains by itself most of the perceptual information in the image. Due to those facts, recently, it has been considered to use the W channel as the major channel in the CFA pattern, and many different RGBW CFAs have been proposed. For example, Figure 2b shows the RGBW CFA proposed in [22].

Compared with other RGBW CFAs [22,23,25] where the W pixels occupy about 50% of the CFA pattern, the proportion of the W channel in the proposed CFA pattern is much higher. This is possible because we use a color interpolation method based on the colorization technique, which can recover the colors in the large proportion of white pixels.

### 2.2. Levin’s Colorization

**Figure 3:**
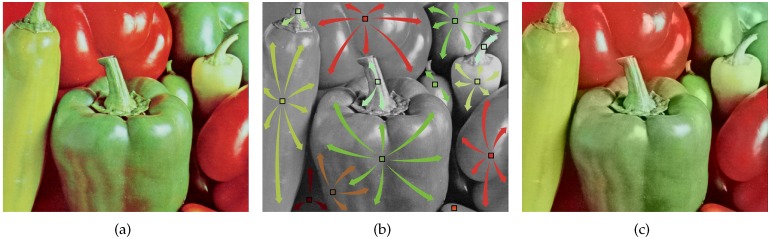
Comparison of (**a**) the original image; (**b**) the gray image with few number of color seeds; and (**c**) the colorized result of (**b**).

The color interpolation method for the proposed CFA is based on the colorization method [30]. In this method, a color image is estimated from a monochrome image with a small number of color seeds which contain the chrominance information as shown in Figure 3. In [30], Levin et al. propose how to formulate the colorization process as an optimization problem. Let *M* denote the number of pixels in the color image, *r* be the pixel position index in raster-scan order (1≤r≤M), y be an M×1 vector denoting the luminance channel, u, an M×1 vector denoting the chrominance channel (U or V) to be solved, and x, an M×1 sparse vector containing the chrominance values only in certain positions (called as the representative pixels) and zeros in all other positions. Furthermore, let Ψ be the set of all *r* where x(r)≠0, and N(r) be the set of the 8-neighborhood pixels of the *r*-th. The colorization process is performed by minimizing the following energy function:

(1) $$J\left(u\right)=\sum_{p\in \Psi }{\left\{x\left(p\right)-u\left(p\right)\right\}}^{2}+\sum_{r\notin \Psi }{\left\{u\left(r\right)-\sum_{s\in N(r)}\omega_{rs}u\left(s\right)\right\}}^{2}.$$

Here, $\omega_{rs}$ is a weighting value defined by

(2) $$\omega_{rs}\propto e^{{\left(y\left(r\right)-y\left(s\right)\right)}^{2}/2\sigma_{r}^{2}}.$$

Define the weighting matrix W of size M×M as follows

(3) $$W\left(r,s\right)=\left\{\begin{array}{c} \omega_{rs}\;\;\;\mathrm{if}\;r\notin \Psi \mathrm{and}\;s\in N(r)\\ 0\;\;\;\mathrm{otherwise}, \end{array}\right.$$

and define A=I−W, where I is an M×M identity matrix. Then, (1) can be expressed as

(4) $$J\left(u\right)={∥x-Au∥}^{2}.$$

The minimizer u of J(u) is the color channel constructed from the small number of color seeds, i.e., from x. The colorization technique brought about the idea that the R, G, and B channels can be reconstructed by a few number of sensed color pixels which contain the true color information, and the full-resolution white channel.

## 3. Proposed Method

### 3.1. Proposed Randomly Sampled RGBW CFA Pattern

**Figure 4:**
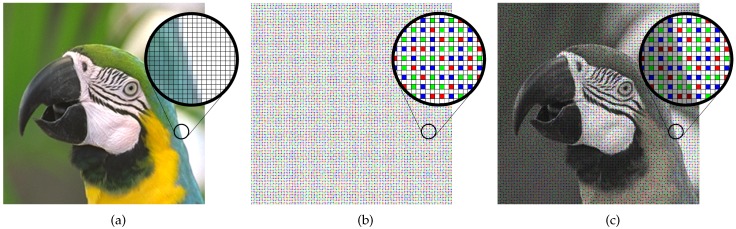
(**a**) Original image; (**b**) randomly sampled RGBW CFA pattern; (**c**) sampled image using the pattern in (**b**) pattern on (**a**).

The proposed RGBW CFA pattern differs from other RGBW patterns in two aspects: it has a larger ratio of W pixels, and the positions of the R, G, and B pixels are random. Figure 4 shows the proposed randomly sampled RGBW pattern. pattern is 75%, and thus receives more light energy than other RGBW CFA patterns. This makes it stronger against the noise. Furthermore, since the W channel corresponds to the luminance channel which determines the resolution of the color image, a high resolution image can be obtained with the proposed CFA. The remaining 25% area of the CFA is composed of RGB pixels, which are randomly distributed over the pattern. The reason that we use a random RGB pixel pattern rather than a periodic one is that the aliasing artifact in the color channels can be reduced. In other RGBW CFAs, the periodic RGBW pattern is converted into the color image by directional interpolation. In this case, some pixels with certain directions cannot be included in the interpolation process, which results in the aliasing artifact.

**Figure 5:**
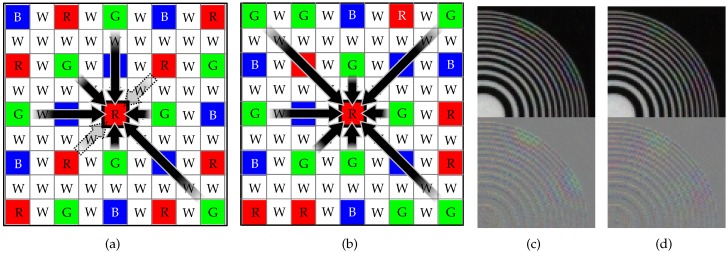
The G pixel estimation at the position of an R pixel with (**a**) a periodic RGBW CFA; and (**b**) a random patterned RGBW CFA; (**c**) Color interpolation result with pattern shown in (**a**); (**d**) Color interpolation result with pattern shown in (**b**).

Figure 5a shows the case of estimating the G channel value at an R pixel in a RGBW pattern which has same proportion of R, G, B, and W pixels, but whose R, G, and B pixels are periodically distributed over the pattern. Here, no G pixels are in the lower-left to upper-right diagonal direction. Therefore, the true G values in this direction cannot be referenced. As a result, aliasing artifact occurs if high frequency components of the G channel exist in this direction. Figure 5c shows the color interpolation result of the RGBW pattern in 5a, where the aliasing artifact can be observed in the upper right and lower left areas. In comparison, the proposed random RGBW pattern has G pixels in all directions, and therefore, the R pixel in Figure 5b can refer to those pixels in estimating the G channel value. Furthermore, the method in referring to those pixels is non-directional, i.e., based on a diffusion method as will be explained later. As a result, the aliasing artifact in the upper-right and lower-left areas is reduced as compared to the periodically sampled RGBW CFA, as can be seen in Figure 5d.

### 3.2. W Channel Interpolation

Most of the demosaicing methods for the Bayer CFA pattern first interpolate the G channel, since it contains most of the spatial information. The information of the interpolated G channel is then used in the subsequent R and B channel interpolation by referring to the inter-channel spectral correlations. Meanwhile, with conventional RGBW CFAs, the RGBW pattern is first converted to the RGB Bayer pattern, and then to the color image. Therefore, the demosaicing method is similar to the one with Bayer CFA pattern, including only an extra process of converting the RGBW pattern to the RGB pattern.

**Figure 6:**
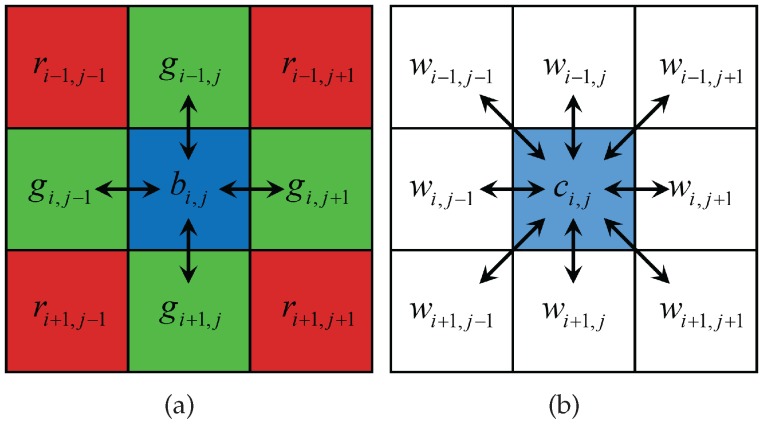
3 × 3 windows of (**a**) Bayer CFA pattern and (**b**) the proposed RGBW CFA pattern.

In comparison, with the proposed method, we reconstruct all the W pixels first, and then recover the colors by the colorization-based interpolation. This is due to the fact that the W channel has a higher spectral correlation with the R and B channels than the G channel, and therefore, is more suitable as a reference channel for recovering the color information. Furthermore, only 25% of the W channel has to be recovered with the proposed RGBW pattern, thus the recovery becomes highly reliable. Figure 6 shows a 3 × 3 local region of the RGBW CFA pattern where the W value of the central pixel has to be estimated. Here, *w* denotes the W channel, and *c* denotes one of the primary color channels, i.e., c∈{r,g,b}. With the Bayer CFA pattern, only four pixels, i.e., the pixels in the horizontal and vertical directions, can be referred to interpolate the missing G value. In comparison, the 8-neighborhood pixels can be used to estimate the missing W pixel values ($\hat{w}$) with the proposed RGBW CFA pattern. Let {i,j} denote the coordinates of the missing W pixel, N(i,j) represent the set of the 8 neighborhood of {i,j}, and {u,v} be the coordinates of the pixels belonging to N(i,j). We define a weighting function $\alpha_{u,v}$ which determines how much the white pixel value in position {u,v}, i.e., $w_{u,v}$, should contribute to the reconstruction of the missing value at {i,j}, i.e., $w_{i,j}$ as follows:

(5) $$\alpha_{u,v}=\frac{1}{\beta_{i,j}\Delta_{u,v}^{inter}+\Delta_{u,v}^{intra}},\;\;\forall u,v\in N\left(i,j\right).$$

Here, $\alpha_{u,v}$ consists of two terms, the inter-channel term $\Delta_{u,v}^{inter}$ and the intra-channel term $\Delta_{u,v}^{intra}$, where $\beta_{i,j}$ acts as a balance between these two terms. Then, reconstruction of $\hat{w}$ is performed by the following equation:

(6) $${\hat{w}}_{i,j}=\frac{\sum_{u,v\in N(i,j)}\alpha_{u,v}w_{u,v}}{\sum_{u,v\in N(i,j)}\alpha_{u,v}}.$$

Equation (6) is a weighted interpolation, where the weights are $\alpha_{u,v}$. As will be seen later, the term $\beta_{i,j}$ in (5) is designed to have a large value in achromatic regions, and a small value in colorful regions. If the difference between $w_{u,v}$ and $w_{i,j}$ is large, this means that the pixels {u,v} and {i,j} belong to different regions in the image, and therefore, the value $w_{u,v}$ should not contribute to the reconstruction of $w_{i,j}$. Therefore, the weight $\alpha_{u,v}$ should be inversely proportional to the difference between $w_{u,v}$ and $w_{i,j}$.

In achromatic regions, the color value at {i,j} itself is similar to the missing value $w_{i,j}$, and thus, the difference between $w_{i,j}$ and $w_{u,v}$ can be approximately measured by the difference between $c_{i,j}$ and $w_{u,v}$, where $c_{i,j}$ refers to one of the primary color values at {i,j}. Therefore, the inter-channel term is defined as

(7) $$\Delta_{u,v}^{inter}=\left|c_{i,j}-w_{u,v}\right|,\;\;\forall u,v\in N\left(i,j\right).$$

**Figure 7:**
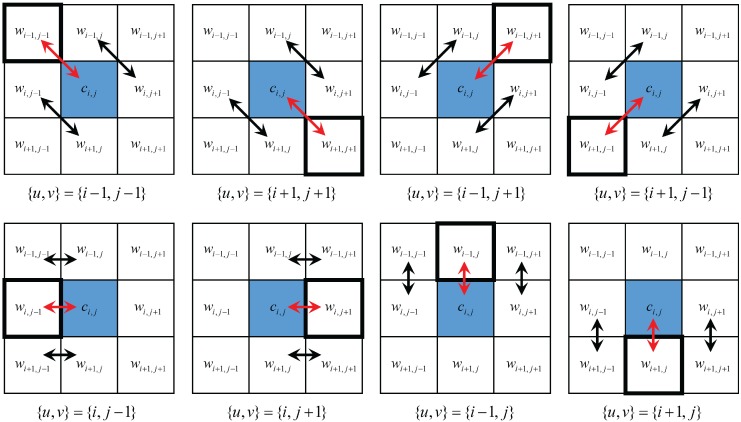
Showing the pixels involved in the calculation of the inter-channel and the intra-channel terms for the 8-neighborhood directions. Red arrows: differences considered in the inter-channel terms $\Delta_{u,v}^{inter}$. Black arrows: differences considered in the intra-channel terms $\Delta_{u,v}^{intra}$.

On the contrary, in colorful regions, $c_{i,j}$ is not similar to $w_{i,j}$, and therefore, the difference between $w_{u,v}$ and $w_{i,j}$ should be measured by the differences of adjacent pixels. For example, if {u,v}={i−1,j−1}, the difference between $w_{i,j}$ and $w_{u,v}$ is measured by the average of $\left|w_{i,j-1}-w_{i+1,j}\right|$ and $\left|w_{i-1,j}-w_{i,j+1}\right|$ as illustrated in the top left image in Figure 7.

The intra-channel terms $\Delta_{u,v}^{intra}$ for different positions of {u,v} in N(i,j) are defined respectively as

(8) $$\begin{array}{c} \Delta_{u=i-1,v=j-1}^{intra}=\left(|w_{i-1,j}-w_{i,j+1}\right|+\left|w_{i,j-1}-w_{i+1,j}|\right)/2,\;\;\;\Delta_{u=i,v=j-1}^{intra}=\left(|w_{i-1,j-1}-w_{i-1,j}\right|+\left|w_{i+1,j-1}-w_{i+1,j}|\right)/2,\\ \Delta_{u=i-1,v=j+1}^{intra}=\left(|w_{i-1,j}-w_{i,j-1}\right|+\left|w_{i,j+1}-w_{i+1,j}|\right)/2,\;\;\;\Delta_{u=i,v=j+1}^{intra}=\left(|w_{i-1,j+1}-w_{i-1,j}\right|+\left|w_{i+1,j+1}-w_{i+1,j}|\right)/2,\\ \Delta_{u=i+1,v=j-1}^{intra}=\left(|w_{i-1,j}-w_{i,j-1}\right|+\left|w_{i,j+1}-w_{i+1,j}|\right)/2,\;\;\;\Delta_{u=i-1,v=j}^{intra}=\left(|w_{i-1,j-1}-w_{i,j-1}\right|+\left|w_{i-1,j+1}-w_{i,j+1}|\right)/2,\\ \Delta_{u=i+1,v=j+1}^{intra}=\left(|w_{i-1,j}-w_{i,j+1}\right|+\left|w_{i,j-1}-w_{i+1,j}|\right)/2,\;\;\;\Delta_{u=i+1,v=j}^{intra}=\left(|w_{i+1,j-1}-w_{i,j-1}\right|+\left|w_{i+1,j+1}-w_{i,j+1}|\right)/2. \end{array}$$

The value $\beta_{i,j}$ which determines whether the pixel {i,j} belongs to an achromatic region or a colorful region is defined as

(9) $$\beta_{i,j}=\left\{\begin{array}{cc} 0.5 & \mathrm{if}m_{i,j}^{inter}\leq m_{i,j}^{intra}\\ 2 & \mathrm{if}m_{i,j}^{inter}\geq 2m_{i,j}^{intra}\\ \frac{1.5(m_{i,j}^{inter}-m_{i,j}^{intra})}{m_{i,j}^{intra}}+0.5 & \mathrm{otherwise}, \end{array}\right.$$

and has a large value in achromatic regions and a small value in colorful regions. Here, $m_{i,j}^{inter}$ and $m_{i,j}^{intra}$ are the minimum values related with $\Delta_{u,v}^{inter}$ and $\Delta_{u,v}^{intra}$, and are defined as

(10) $$\begin{array}{c} m_{i,j}^{inter}=\min\left(\Delta_{i-1,j-1}^{inter}+\Delta_{i+1,j+1}^{inter},\Delta_{i-1,j+1}^{inter}+\Delta_{i+1,j-1}^{inter},\Delta_{i,j-1}^{inter}+\Delta_{i,j+1}^{inter},\Delta_{i-1,j}^{inter}+\Delta_{i+1,j}^{inter}\right)\\ m_{i,j}^{intra}=\min\left(\Delta_{i-1,j-1}^{intra}+\Delta_{i+1,j+1}^{intra},\Delta_{i-1,j+1}^{intra}+\Delta_{i+1,j-1}^{intra},\Delta_{i,j-1}^{intra}+\Delta_{i,j+1}^{intra},\Delta_{i-1,j}^{intra}+\Delta_{i+1,j}^{intra}\right). \end{array}$$

### 3.3. Primary Color Channel Interpolation

As described in Section 3.1, the number of color seeds, i.e., pixels which are sensing the primary colors, is very small, and their positions are randomly distributed. After the W channel is fully interpolated with the aforementioned method, the primary color channels can be obtained with a colorization scheme. However, in low light conditions, the primary color channels are more degraded by the noise than the W channel. Therefore, in this work, we design a colorization matrix which simultaneously diffuse the original color seeds to other pixels and remove the noise in the original color seeds.

Let w and c represent the lexicographically ordered vectors corresponding to the 2-dimensional images *w* and *c*, i.e.,

(11) $$w={\left[w_{1,1},w_{2,1},\ldots ,w_{N_{r},1},w_{1,2},\ldots ,w_{1,N_{c}},\ldots ,w_{N_{r},N_{c}}\right]}^{T},$$

and

(12) $$c={\left[c_{1,1},c_{2,1},\ldots ,c_{N_{r},1},c_{1,2},\ldots ,c_{1,N_{c}},\ldots ,c_{N_{r},N_{c}}\right]}^{T},$$

where $N_{r}$ and $N_{c}$ denote the height and the width of the image. Here, c represents the color channel which we want to recover from the sensed RGBW pattern image.

While other sophisticated residual images such as that proposed in [11] can also be used, we use a simple color difference channel. The color difference channel $u_{c}$ between the primary color channel c∈{r,g,b} and the W channel is defined as:

(13) $$u_{c}=c-w.$$

Only 25% of the components in the $u_{c}$ vector are sensed by the proposed RGBW CFA, and the problem of the primary color channel reconstruction, i.e., the color channel interpolation, is to recover the true $u_{c}$ vector with proper color components at every position. We define by $x_{c}$ the vector which contains the $u_{c}$ values only at the positions where the corresponding c value is sensed by the proposed RGBW CFA and has zero values at all other positions, i.e.,

(14) $$x_{c}\left(m\right)=\left\{\begin{array}{cc} c\left(m\right)-\hat{w}\left(m\right) & \mathrm{if}m\in \Psi_{c}\\ 0 & \mathrm{otherwise}, \end{array}\right.$$

where $\hat{w}$ is the full W channel reconstructed by the method in Section 3.2, *m* is the position index of the pixel in the lexicographically ordered vector $x_{c}$, and $\Psi_{c}$ represents the set of the pixel positions where the channel *c* value is sensed by the RGBW CFA. In accordance with [30], we call the pixels in the set $\Psi_{c}$ the representative pixels or the color seeds.

The proposed colorization-based color interpolation solves the interpolation problem by minimizing the following functional with respect to $u_{c}$ given $x_{c}$ and $\hat{w}$:

(15) $$J\left(u_{c}\right)=D\left(u_{c}\right)+F\left(u_{c}\right).$$

The functional $J(u_{c})$ consists of two energy terms: the diffusion term $D(u_{c})$ and the noise-suppressing fidelity term $F(u_{c})$. Let *r* denote the position index of the pixels not belonging to $\Psi_{c}$, and N(r) the neighborhood pixels of *r*. The diffusion term is defined as

(16) $$D\left(u_{c}\right)=\sum_{r\notin \Psi_{c}}{\left\{u_{c}\left(r\right)-\sum_{s\in N(r)}\epsilon_{rs}u_{c}\left(s\right)\right\}}^{2}.$$

**Figure 8:**
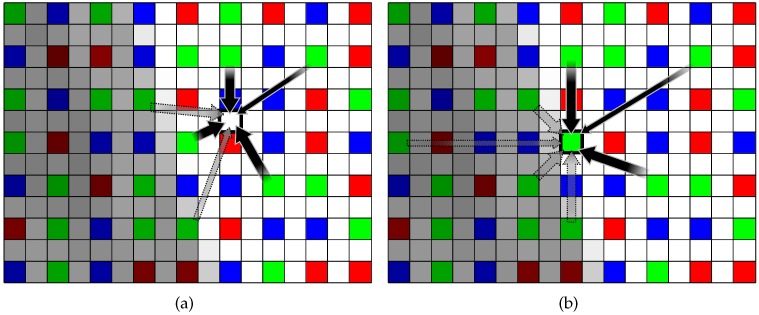
(**a**) The diffusion and (**b**) the noise suppression process of the colorization matrix in the edge region of the RGBW sampled image. The contribution of the pixels in a region different from that in which the pixel under consideration lies is weak as visualized by the faint colored arrows.

The minimization of $D(u_{c})$ can be seen as weighted diffusion shown in Figure 8a, where the amount of diffusion between *r* and *s* is determined by the weight $\epsilon_{rs}$. The weight $\epsilon_{rs}$ is controlled by an edge directional ellipsoidal kernel as will be explained later. Let *p* denote the position index of the pixels belonging to $\Psi_{c}$, and N(p) the neighborhood pixels of *p*. The noise-suppressing fidelity term $F(u_{c})$ is defined as

(17) $$F\left(u_{c}\right)=\sum_{p\in \Psi_{c}}{\left[u_{c}\left(p\right)-x\left(p\right)+\gamma \left\{u_{c}\left(p\right)-\sum_{q\in N(p)}\epsilon_{pq}u_{c}\left(q\right)\right\}\right]}^{2}.$$

It tries to not only make $u_{c}$ similar to $x_{c}$, but also subtract the weighted Laplacian of $u_{c}$, i.e., $\gamma \{\sum_{q\in N(p)}\epsilon_{pq}u_{c}\left(q\right)-u_{c}\left(p\right)\}$ from $u_{c}$ at the seed pixel. It is different from the classical cost function defined as follow:

(18) $$J_{old}\left(u_{c}\right)=\sum_{p\in \Psi_{c}}{\left[u_{c}\left(p\right)-x_{c}\left(p\right)\right]}^{2}+\gamma \sum_{p}{\left[\left\{u_{c}\left(p\right)-\sum_{q\in N(p)}\epsilon_{pq}u_{c}\left(q\right)\right\}\right]}^{2}.$$

Minimizing (18), the neighborhood pixels tend to follow the noisy seed pixels and result in low frequency noise.

The first term in (17) preserves the fidelity of $u_{c}\left(p\right)$ to the sensed value at *p*, i.e., $x_{c}\left(p\right)$, while the second term tries to smooth it as a weighted average of the values of the neighborhood pixels as shown in Figure 8b, where the weights $\epsilon_{pq}$ are obtained in the same manner as $\epsilon_{rs}$ as will be explained later. The second term is necessary because, unlike the original colorization problem, the color seeds in the proposed method are degraded by the noise, especially in low light condition. This leads to spotted color noise after applying the colorization-based interpolation process. The value γ controls the balance between the two terms, thus controlling the degree of diffusion. When γ is large, the values of $u_{c}$ at the position of the color seeds are influenced much by the nearby color values, whereas if γ=0, they are not influenced at all by the nearby values. Here, γ is proportional to the noise variance. Let $\sigma_{n}^{2}$ denote the noise variance and τ represent a control constant, γ is defined as

(19) $$\gamma =\tau \sigma_{n}^{2}.$$

The noise variance $\sigma_{n}^{2}$ can be estimated by calculating the variance of the flat region in the image. In [30], the weight parameters were determined based on the differences of the luminance values between adjacent pixels, so that the color seeds were easily spread out in flat regions, while not in edge regions. In low light conditions, however, the difference between adjacent pixel values could not be estimated correctly due to the noise. As some edge regions were weakened by the noise, the color diffused across the edge region which results in the wash-out of the colors in edge regions. As the edge could not be determined exactly using the derivative at the current position only, the derivatives in a neighborhood region should be considered together to determine the edge direction.

The weight parameter is designed to take into account the directional tendency of adjacent pixels [31,32]. In order to estimate the tendency of the edge direction, the covariance matrix C is determined in a small region R(m) centered at *m*

(20) $$C=\left[\begin{array}{cc} \sum_{n\in R(m)}{\hat{w}}_{v}\left(n\right){\hat{w}}_{v}\left(n\right) & \sum_{n\in R(m)}{\hat{w}}_{v}\left(n\right){\hat{w}}_{h}\left(n\right)\\ \sum_{n\in R(m)}{\hat{w}}_{v}\left(n\right){\hat{w}}_{h}\left(n\right) & \sum_{n\in R(m)}{\hat{w}}_{h}\left(n\right){\hat{w}}_{h}\left(n\right) \end{array}\right],$$

where ${\hat{w}}_{v}$ and ${\hat{w}}_{h}$ represent the derivatives of $\hat{w}$ in the vertical and the horizontal directions, respectively. Let $d_{mn}={\left[v_{mn},h_{mn}\right]}^{T}$ be a pointing vector, where $v_{mn}$ and $h_{mn}$ are the vertical and the horizontal distances between *m*, the current pixel position, and *n*, a neighborhood pixel of *m*. The weight value at *n* is calculated as

(21) $$\epsilon_{mn}^{\prime }=\exp\left\{-\lambda d_{mn}^{T}Cd_{mn}\right\}.$$

**Figure 9:**
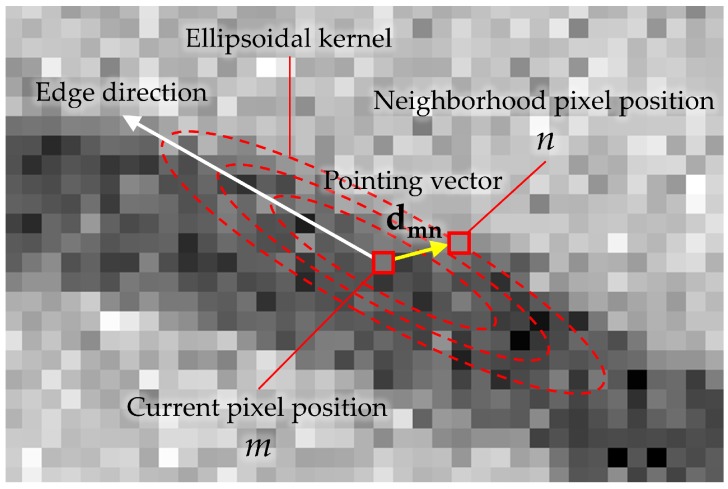
The noisy edge region and an ellipsoidal kernel for the pixel position *m*. The major axis of the ellipsoidal kernel is parallel to the edge direction.

It is derived from an ellipsoidal kernel function (Figure 9) which has a large value if *n* lies at the center of the kernel and a small value if *n* is far from the center. Here, λ represents a parameter which controls the smoothness of the kernel. Furthermore, $\epsilon_{mn}^{\prime }$ is large if *n* lies in a direction orthogonal to the edge, and small if *n* lies in the direction of the edge. Therefore, $\epsilon_{mn}^{\prime }$ can be used as a measure whether a pixel *n* lies along or across an edge. For the weights in (16) and (17), we use a normalized version of $\epsilon_{mn}^{\prime }$, i.e., $\epsilon_{mn}=\frac{1}{k}\epsilon_{mn}^{\prime }$, where *k* is the normalizing factor.

The cost function defined in (15) can be re-written in matrix form as

(22) $$J\left(u_{c}\right)={∥Du_{c}∥}^{2}+{∥Fu_{c}-x_{c}∥}^{2},$$

where the diffusion matrix D which spreads the color seeds is defined as

(23) $$D\left(r,s\right)=\left\{\begin{array}{cc} 1 & \mathrm{if}\;r\notin \Psi_{c}\mathrm{and}\;s=r\\ -\epsilon_{rs} & \mathrm{else}\;\mathrm{if}\;r\notin \Psi_{c}\mathrm{and}\;s\in N\left(r\right)\\ 0 & \mathrm{otherwise}. \end{array}\right.$$

The noise-suppressing fidelity matrix F is defined as

(24) $$F\left(p,q\right)=\left\{\begin{array}{cc} 1+\gamma & \mathrm{if}\;p\in \Psi_{c}\mathrm{and}\;q=p\\ -\gamma \epsilon_{pq} & \mathrm{else}\;\mathrm{if}\;p\in \Psi_{c}\mathrm{and}\;q\in N\left(p\right)\\ 0 & \mathrm{otherwise}. \end{array}\right.$$

Solving (22), the color difference channel can be estimated as follows:

(25) $${\tilde{u}}_{c}={\left(D^{T}D+F^{T}F\right)}^{-1}F^{T}x_{c}.$$

By using the estimated color difference channel ${\hat{u}}_{c}$, the primary color channel can be obtained as

(26) $$\tilde{c}={\tilde{u}}_{c}+\hat{w}.$$

### 3.4. Post Processing

The color channels $\tilde{c}\in \left\{\tilde{r},\tilde{g},\tilde{b}\right\}$ reconstructed by the method explained in Section 3.3 suffer from low frequency noise. This is due to the fact that the color seeds from which they are reconstructed contain noise themselves. In this section, we propose a post-process which subtracts the low frequency noise $n_{LF}$ from $\tilde{c}$ to obtain a noise removed color channel $\hat{c}$:

(27) $$\hat{c}=\tilde{c}-n_{LF}.$$

Here, the low frequency noise $n_{LF}$ is estimated by subtracting $\hat{w}$ obtained in Section 3.2 from $\tilde{w}$ which denotes a noisy W channel to be constructed as follow:

(28) $$n_{LF}=\tilde{w}-\hat{w}.$$

This is based on the assumptions that the low frequency noise contained in $\tilde{c}$ is highly correlated with the noise in $\tilde{w}$, and that $\hat{w}$ is free from this low frequency noise. The first assumption is satisfied since the noisy $\tilde{w}$ is constructed by a linear combination of the noisy color channels:

(29) $$\tilde{w}=\lambda_{r}\tilde{r}+\lambda_{g}\tilde{g}+\lambda_{b}\tilde{b}+\lambda_{1}.$$

Here, $\lambda_{r}$, $\lambda_{g}$, $\lambda_{b}$, and $\lambda_{1}$ are linear coefficients which are obtained by the following $L_{2}$ norm minimization with respect to multiple arguments $\lambda_{r}$, $\lambda_{g}$, $\lambda_{b}$, and $\lambda_{1}$:

(30) $$\lambda_{r},\lambda_{g},\lambda_{b},\lambda_{1}=\underset{\lambda_{r},\lambda_{g},\lambda_{b},\lambda_{1}}{\mathrm{argmin}}{∥\hat{w}-\left(\lambda_{r}\tilde{r}+\lambda_{g}\tilde{g}+\lambda_{b}\tilde{b}+\lambda_{1}\right)∥}_{2}^{2}.$$

**Figure 10:**
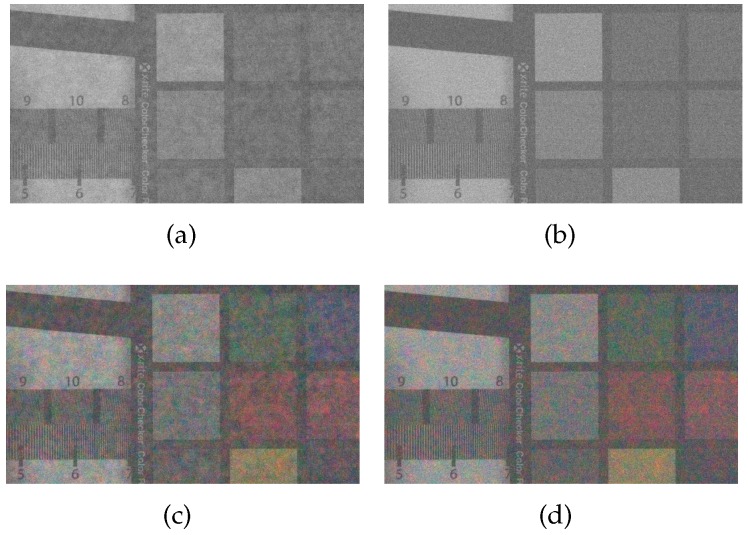
(**a**) The reconstructed W channel $\tilde{w}$ by (29); (**b**) the reconstructed W channel $\hat{w}$ after applying the post-process in Section 3.2; (**c**) the reconstructed color channel $\tilde{c}$ obtained by the method in Section 3.3; and (**d**) the noise removed color channel $\hat{c}$ after applying the post-process in Section 3.2.

This is a multiple regression model [33] which makes the $L_{2}$ norm difference between $\hat{w}$ and are $\tilde{w}$ as small as possible, thus resulting in the coefficients $\lambda_{r}$, $\lambda_{g}$, $\lambda_{b}$ and $\lambda_{1}$ that the overall brightness of $\tilde{w}$ becomes similar to $\hat{w}$. This is similar to the guided filter approach proposed in [34], but instead of a single parameter we use multiple parameters, and unlike the guided filter which tries to obtain a noise-free channel, the purpose is to obtain a noisy white image $\tilde{w}$ which is used afterwards to eliminate the noise in the reconstructed noisy color channels. The constructed channel $\tilde{w}$ contains the same low frequency noise as in $\tilde{c}$ since it is constructed from it. The second assumption is satisfied since $\hat{w}$ is constructed from the sensed W channel, which has a larger SNR value than those of the color channels. Using the linear least square method, (30) can be solved as follow:

(31) $$\left[\begin{array}{c} \lambda_{r}\\ \lambda_{g}\\ \lambda_{b}\\ \lambda_{1} \end{array}\right]={\left[\begin{array}{cccc} \mu \{\tilde{r}\} & \mu \{\tilde{g}\} & \mu \{\tilde{b}\} & 1\\ \mu \{\tilde{r}\circ \tilde{r}\} & \mu \{\tilde{g}\circ \tilde{r}\} & \mu \{\tilde{b}\circ \tilde{r}\} & \mu \{\tilde{r}\}\\ \mu \{\tilde{r}\circ \tilde{g}\} & \mu \{\tilde{g}\circ \tilde{g}\} & \mu \{\tilde{b}\circ \tilde{g}\} & \mu \{\tilde{g}\}\\ \mu \{\tilde{r}\circ \tilde{b}\} & \mu \{\tilde{g}\circ \tilde{b}\} & \mu \{\tilde{b}\circ \tilde{b}\} & \mu \{\tilde{b}\} \end{array}\right]}^{-1}\left[\begin{array}{c} \mu \{\hat{w}\}\\ \mu \{\hat{w}\circ \tilde{r}\}\\ \mu \{\hat{w}\circ \tilde{g}\}\\ \mu \{\hat{w}\circ \tilde{b}\} \end{array}\right],$$

where $a\in \{\tilde{r},\tilde{g},\tilde{b},\hat{w}\}$, μ{a} denotes the mean of a vector a, and ∘ denotes the entrywise product operator. Figure 10a–d show $\tilde{w}$, $\hat{w}$, $\tilde{c}$, and $\hat{c}$, respectively. As can be seen in Figure 10d, the low frequency noise in $\hat{c}$ is quite reduced.

## 4. Experimental Results

We compared the quality of the demosaicing result of the proposed method based on the proposed RGBW CFA pattern with the demosaicing results of other methods based on other CFA patterns. Experimental results show that in low light condition, spatial information which cannot be obtained by the Bayer CFA pattern using demosaicing method can be obtained by the proposed method. Furthermore, we also show that the proposed method produces results of good quality in sufficient light condition. In order to obtain the original full resolution R, G, B and W channel images, we used a filter-wheel-installed camera, where the wheel contains four different optical filters and is driven by a stepping motor. The four optical filters selectively filter the R, G, B, and W bands. We took four photographs of the same scene with the four different filters to obtain the R, G, B, and W channels. Figure 1 shows the spectral responsibilities of the R, G, B, and W filters. Here, the exposure time was set to 30 ms at 100 lux illumination. Using the full-resolution four channel images, we sampled the R, G, B, and W pixels corresponding to the different patterns, i.e., the Bayer CFA pattern, the Sony RGBW CFA pattern, and the proposed RGBW CFA pattern. For the proposed RGBW CFA pattern, the numbers of the R, G, and B pixels are 306,500, 306,697, and 306,489, respectively, which are not equal since there are randomly sampled, but together they cover 25% of the CFA pattern which has a total of 3,686,400 pixels since the size of the image is 1920×1920. The control constant τ is set to a value around 100.

**Figure 11:**
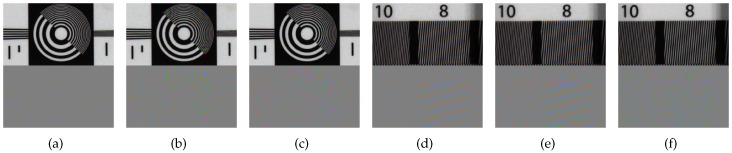
Experimental results and their aliasing artifacts in 100 lux light condition: (**a**,**d**) results using the Bayer CFA; (**b**,**e**) results using the Sony RGBW CFA; and (**c**,**f**) results using the proposed RGBW CFA.

The images in the first column in Figure 11 show the demosaiced results of the Bayer CFA, and those in the second column are the demosaiced results of the Sony RGBW CFA. For the Bayer CFA results, the DLMMSE method [7] was used, while with the Sony RGBW CFA, the RGBW pattern was first converted into the Bayer CFA pattern using the method in [22], and then finally got demosaiced by the DLMMSE method. The images in the third column in Figure 11 show the results of the proposed RGBW CFA which are reconstructed by the proposed colorization-based interpolation method. As described in Section 3.1, the results using the Sony RGBW CFA shows the largest aliasing artifacts due to the extra errors in the conversion from the RGBW to the Bayer CFA. The Bayer CFA based conversion shows less aliasing artifacts in the circle region than the proposed RGBW CFA based conversion in the first row of Figure 11. However, in the vertical stripe region, the result with the proposed method is better than those using other methods in terms of both the aliasing and the noise artifacts as can be seen in the second row of Figure 11.

**Figure 12:**
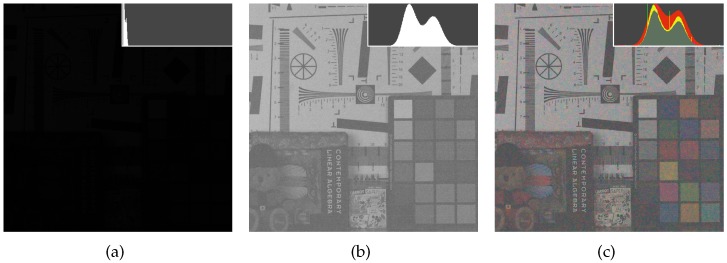
Images and their histograms: (**a**) the acquired image with the proposed RGBW CFA pattern before white balancing; (**b**) the pattern image after white balancing; and (**c**) color image from reconstructed from (**b**).

Next, for an assessment in low light condition, we obtained the full-resolution R, G, B, and W images using the four different optical filters with an exposure time of 1/60 s at 1 lux illumination. After that, we sampled the R, G, B, and W pixels corresponding to the Bayer CFA and the proposed RGBW CFA patterns. The image acquired at 1 lux illumination is very dark as shown in Figure 12a. The energy levels of the R, G, B, and W channels are different due to the different spectral responses of illumination and sensor sensitivity. Therefore, we first multiplied a large number (420) to the pixels of the acquired image to stretch the ranges in the pixel values, and then applied a white balancing as a pre-process to obtain Figure 12b. We performed the gray world method [35] which is one of the simplest white balancing method, but other methods [35,36,37,38,39,40] can be applied as well with little difference in the reconstruction performance. Figure 12b illustrates the result and its corresponding histogram after rescaling the intensity range and applying white balancing to the acquired image. The color interpolation is then performed on this white-balanced image, resulting in the color image shown in Figure 12c.

**Figure 13:**
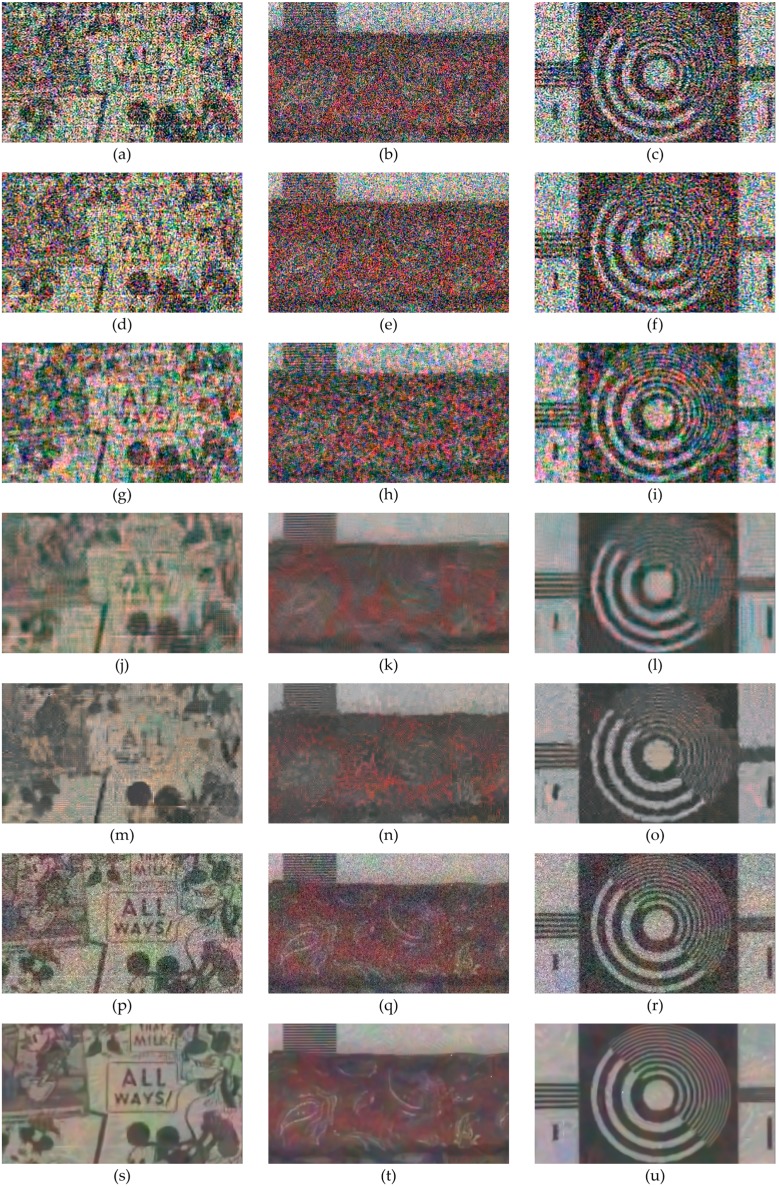
Experimental results with 1 lux low light condition. (**a–c**): results using the Bayer CFA with DLMMSE [7], (**d–f**): results using the Bayer CFA with RI [11], (**g–i**): results using the Sony RGBW CFA [22], (**j–l**): results using the denoised Bayer CFA with Akiyama [28], (**m–o**): results using the denoised Bayer CFA with BM3D-CFA [29], (**p–r**): results using the proposed RGBW CFA, and (**s–u**): results using the proposed RGBW CFA + BM3D.

In Figure 13, we compared the results of the proposed method with the images reconstructed from the Bayer CFA sampled images using the state-of-the-art Bayer denoising methods [28,29]. The methods in [28,29] utilize variations of the BM3D(Block-matching and 3D filtering) method for the denoising of the Bayer-patterned image. We show that in low illumination, the proposed method results in better reconstructed images even without any extra sophisticated denoising methods as in [28,29] which is due to the nature of the proposed CFA having a very large number of W pixels. In the case with 20 dB and 23 dB noise simulations, the BM3D denoising removes the noise well, resulting in a reconstructed image of higher SNR values than that obtained by the proposed method not using a sophisticated denoising method. To make a fair comparison, we also applied the BM3D method to the W channel as a post-denoising method. We cannot apply the exactly same BM3D method as in [28,29] to our method, because they are mainly designed for the Bayer pattern, which is different from the proposed RGBW pattern. Therefore, we applied the BM3D denoising [41] on the W channel, and afterward used it as the reference channel. We call the modified proposed method with BM3D denoising the ‘proposed + BM3D’ method. The input parameter for the denoising methods in [28,29,41] is the level of noise, for which we give the standard deviation of the noise. The standard deviation of noise in the RGB channels is 0.25 (40 in 8 bit system), and that of the noise in the W channel is 0.06 (16 in 8 bit system) in 1 lux low light condition.

Figure 13 compares the results reconstructed by the proposed RGBW CFA, the Bayer CFAs [7,11], the denoised Bayer CFAs [28,29], and the Sony RGBW CFA [22]. It can be seen in Figure 13, that the results obtained by the proposed method exhibit superior high frequency information compared with other results. Moreover, the objects are better identified, and the image details, such as letters and lines, are more distinguished, especially in Figure 13p compared with Figure 13a,d,g,j,m. Figure 13p has also less noise than Figure 13a,d,g in flat regions. The results with Bayer CFA denoising show a lot of noise removal in flat regions, but also have lost important detail information. The ‘proposed + BM3D’ method shows the best result both in flat and detailed regions which can be observed in Figure 13s,t,u. Here, the denoising is done on the reconstructed W channel, which is then used for the colorization of the color seeds.

For objective assessments, we used the Kodak 24 test images of size 512 × 768 and measured the FSIM [42] and PSNR of the reconstructed images, where the PSNR is given by

(32) $$PSNR=10\times \log_{10}\left\{1/\mathrm{MSE}\right\}.$$

We made two sets of test images, one containing 20 dB noise, and the other 23 dB noise, where the noise follows a Poisson distribution as is the case in low illumination. The noise was added to the R, G, and B channels, respectively. The standard deviations of the noises in the RGB channels are 0.1 (26 in 8 bit system) and 0.07 (18 in 8 bit system), respectively. The standard deviations of the noises in the W channel are $0.1/\sqrt{3}=0.06$ (16 in 8 bit system) and $0.07/\sqrt{3}=0.04$ (10 in 8 bit system) in 20 dB and 23 dB, respectively. This is due to the fact that the W channel is a superposition of the R, G, and B channels. The standard deviations of the noises go in as the parameters to the BM3D denoising methods. After that, the pixels were sampled according to the Bayer CFA, the Sony RGBW CFA, and the proposed RGBW CFA pattern, respectively, to generate the CFA pattern images. For the proposed RGBW CFA, the numbers of the color seeds for the R, G, and B channels are 32,456, 32,550, and 32,659, respectively, which together sums up to 25% of the whole pixels (512×768=393,216), and the rest of the pixels (75%) are W pixels. Then, the images sampled by the proposed RGBW CFA were reconstructed by the colorization-based method, while the images sampled by Bayer CFA are demosaiced by the DLMMSE and RI methods. We also added the results using the methods in [28,29]. As shown in Table 1, Table 2, Table 3 and Table 4, the color images reconstructed by the proposed method show significant improvements compared with the results of the Bayer CFA and Sony RGBW CFA. Bold text in the tables are the optimum results for each Kodak images.

**Figure 14:**
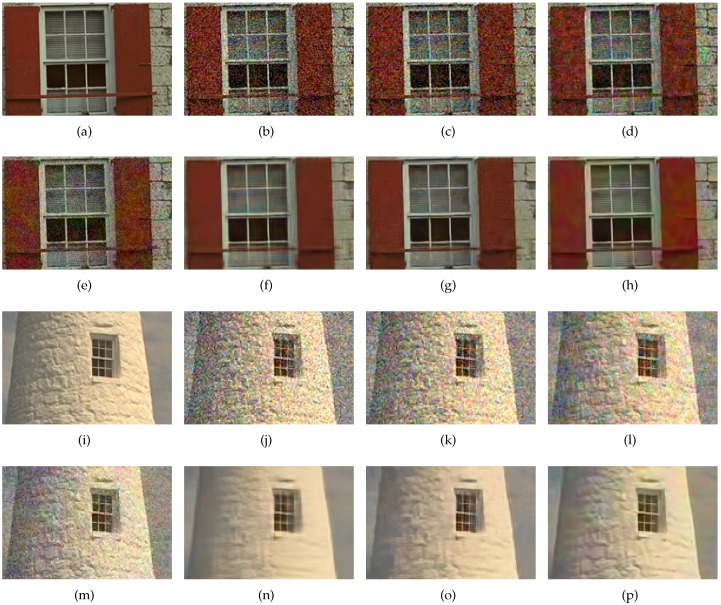
Experiment results of Kodak images with 20dB noise. (**a**,**i**): original images; (**b**,**j**): results using the Bayer CFA with DLMMSE [7]; (**c**,**k**): results using the Bayer CFA with RI [11]; (**d**,**l**): results using the Sony RGBW CFA [22]; (**e**,**m**): results using the proposed RGBW CFA; (**f**,**n**): results using the denoised Bayer CFA with Akiyama [28]; (**g**,**o**): results using the denoised Bayer CFA with BM3D-CFA [29]; and (**h**,**p**): results using the proposed RGBW CFA + BM3D.

**Figure 15:**
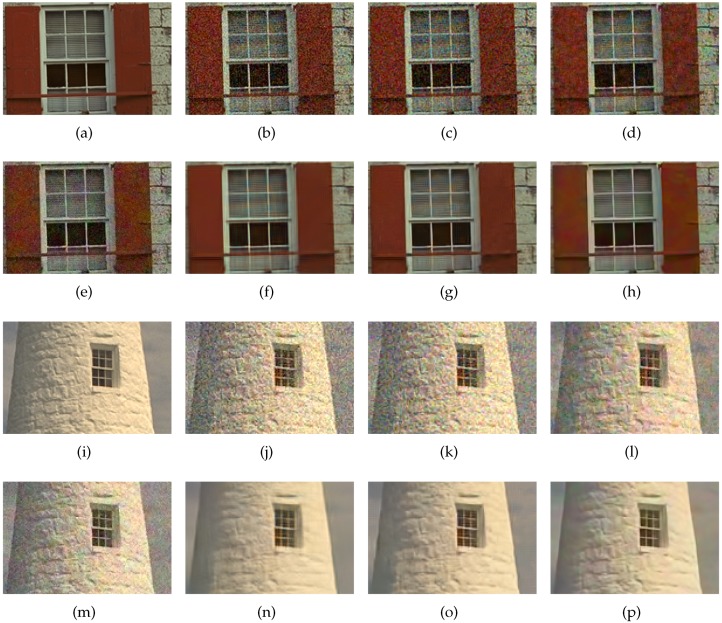
Experiment results of Kodak images with 23dB noise. (**a**,**i**): original images; (**b**,**j**): results using the Bayer CFA with DLMMSE [7]; (**c**,**k**): results using the Bayer CFA with RI [11]; (**d**,**l**): results using the Sony RGBW CFA [22]; (**e**,**m**): results using the proposed RGBW CFA; (**f**,**n**): results using the denoised Bayer CFA with Akiyama [28]; (**g**,**o**): results using the denoised Bayer CFA with BM3D-CFA [29]; and (**h**,**p**): results using the proposed RGBW CFA + BM3D.

The PSNR is improved by an average of 3.2934 dB, 3.2938 dB, and 0.8639 dB, compared to the results of the DLMMSE, RI, and Sony RGBW CFA, respectively, with 20 dB noise. In the case of 23 dB noise, the PSNR is improved by an average of 2.7725 dB, 2.3157 dB, and 0.5639 dB, compared to the results of the DLMMSE, RI, and Sony RGBW CFA, respectively. As shown in Figure 14 and Figure 15, the visual qualities of the images obtained by the proposed method are superior to those by the Bayer CFA and Sony RGBW CFA.

The PSNR and FSIM values of the images obtained by combining the BM3D denoising are higher than the those obtained by the proposed method without BM3D denoising. However, the images reconstructed by the ‘proposed + BM3D’ method show higher FSIM values and similar PSNR values compared with the results of [28,29]. As shown in Figure 14h,p and Figure 15h,p, the results of the ‘proposed + BM3D’ show similar noise levels in flat regions, but preserve more detail information. Also the ‘proposed + BM3D’ introduces fewer aliasing artifacts in high frequency regions as shown in the window bars in Figure 14 and Figure 15. The Bayer CFA denoising methods not only remove the noise in the flat regions but also the details in high frequency regions. This is not reflected effectively in the PSNR measure, but in the FSIM measure which reflects the edge fidelity and the color difference consistency, which is the reason that the FSIM values of the ‘proposed + BM3D’ method are higher in all cases.

**Figure 16:**
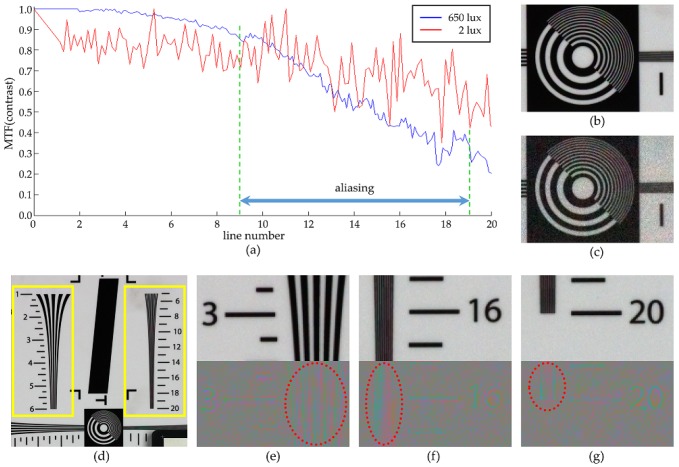
(**a**) MTF graph with high lighting (650 lux, blue line) and low lighting (2 lux, red line) condition; (**b**) reconstruction result with high light condition; (**c**) reconstruction result with low light condition; (**d**) reconstruction result (full image) with high light condition; Showing the region of (**e**) line 3; (**f**) line 16; and (**g**) line 20, with the aliasing artifact in the lower parts.

Figure 16 illustrates the relation between the modulation transfer function(MTF) and the aliasing artifact in the reconstructed images with the proposed method. The value of the horizontal axis in Figure 16a is equal to the line number shown in Figure 16d, which also represents the spatial frequency value. This is due to the fact that the line becomes thinner and denser as the line number increases as can be seen in the yellow boxes in Figure 16d. When the line number is sufficiently low, aliasing is weak as can be seen in Figure 16e. The aliasing artifact is strongest in the region of line 16 (Figure 16f), and again decreases in the region of line 20 due to the lens diffraction (Figure 16g) which also decreases the MTF value. Meanwhile, the MTF is greatly affected by the noise in low light conditions and color artifacts occur due to the diffusion of false signals as can be observed in Figure 16c which is obtained with low lighting, compared to Figure 16b obtained with high lighting.

## 5. Conclusions

In this paper, we proposed a colorization-based demosaicing method which suits well with the proposed RGBW CFA pattern, having a large number of white pixels. Using the proposed demosaicing technique, color images can be reconstructed using a small number of sensed color pixels while the majority of the pixels can sample the white channel, which makes it possible to obtain a reconstructed color image with high SNR value. Compared with the Bayer CFA based results, the image details, such as the letters or lines, are better preserved with the proposed method in low illumination condition. This is important especially for surveillance camera systems, and therefore, the proposed method can find its application in such areas.

## Figures and Tables

**Table 1:** Comparison of the PSNR (dB) values of the Kodak RGB images interpolated by the Bayer CFA, Sony RGBW CFA, denoised Bayer CFA, and proposed RGBW CFA with 20 dB noise levels.

Data	Bayer DLMMSE [7]	Bayer RI [11]	Sony RGBW [22]	Proposed	Akiyama [28]	BM3D-CFA [29]	Proposed + BM3D
1	20.6240	21.0253	22.8337	24.0300	25.7640	26.2185	**26.5624**
2	21.0217	21.5777	24.1440	23.2595	28.7567	**29.0252**	27.2122
3	20.7928	21.5582	23.5414	23.8333	30.6593	**30.8327**	29.2358
4	20.7814	21.4473	23.7901	24.1515	29.3016	**29.5208**	28.4922
5	20.9141	21.3347	22.7660	23.8527	25.2292	**26.0602**	26.0390
6	20.8267	21.2730	22.9500	24.2042	27.0638	27.2829	**27.6730**
7	20.7311	21.3759	23.4218	24.3754	29.4845	**29.9281**	29.4038
8	20.7274	20.9955	22.7796	23.9210	25.4553	26.1903	**26.7617**
9	20.6406	21.3839	23.5115	24.5921	30.5982	**30.7339**	30.2494
10	20.6563	21.3936	23.4530	24.5911	30.0215	30.1505	**30.2441**
11	20.8227	21.3718	23.2586	24.3298	27.6005	28.0054	**28.1005**
12	20.7841	21.4705	23.4702	24.5056	30.5373	**30.6068**	30.0079
13	20.6527	20.8826	21.7919	23.4370	23.9516	24.2884	**24.9340**
14	20.8022	21.2953	23.0835	23.7002	26.4241	**27.0160**	25.8387
15	21.4102	21.8628	23.9531	24.4306	29.1291	**29.3797**	28.2387
16	20.6903	21.3393	23.4063	24.5378	29.4499	**29.5209**	29.0873
17	21.0825	21.6626	23.8226	24.8379	29.2484	29.5851	**29.7193**
18	20.8700	21.4001	22.9114	23.9673	26.0022	**26.5466**	26.5046
19	20.6914	21.2650	23.2757	24.3600	28.6516	**29.0308**	28.7008
20	21.8899	22.3182	24.1087	24.8277	26.9488	27.8986	**28.0698**
21	20.6429	21.2543	23.0521	24.2204	27.4789	27.8329	**28.1341**
22	20.6400	21.2883	23.1219	24.1220	27.8864	**28.2483**	27.8049
23	20.7934	21.5650	23.3896	23.2165	**31.1036**	30.8427	28.3076
24	20.6561	21.1593	22.6258	23.8926	25.9713	26.4484	**26.6763**
Avg	20.8394	21.3959	23.2693	24.1332	28.0299	**28.3831**	27.9999

**Table 2:** Comparison of the PSNR (dB) values of the Kodak RGB images interpolated by the Bayer CFA, Sony RGBW CFA, denoised Bayer CFA, and proposed RGBW CFA with 23 dB noise levels.

Data	Bayer DLMMSE [7]	Bayer RI [11]	Sony RGBW [22]	Proposed	Akiyama [28]	BM3D-CFA [29]	Proposed + BM3D
1	23.4743	23.7315	25.2154	26.1998	27.1647	27.6840	**28.2742**
2	23.7555	24.0597	26.5783	26.0850	29.8295	**30.1677**	29.1778
3	23.6888	24.3995	26.5252	26.5784	31.9909	**32.3835**	31.0654
4	23.6173	24.1784	26.5210	26.3983	30.3262	**30.6601**	30.1931
5	23.6914	24.0412	24.9118	25.8992	27.0623	**27.8761**	27.7638
6	23.6902	24.0157	25.5248	26.3360	28.5588	28.8965	**29.2019**
7	23.6396	24.2284	26.3332	26.7672	31.2039	**31.8072**	31.1182
8	23.4988	23.6250	25.0019	26.0813	27.1062	27.8035	**28.3955**
9	23.5865	24.2659	26.5441	27.0729	32.1003	**32.3720**	31.6528
10	23.6309	24.2935	26.5336	27.1205	31.4922	**31.8247**	31.8130
11	23.6742	24.1131	25.9178	26.5520	28.9809	**29.5593**	29.5306
12	23.6820	24.3276	26.4528	26.8893	31.7944	**32.1896**	31.4717
13	23.4140	23.4268	23.5726	25.1309	25.5672	25.8364	**26.3775**
14	23.5546	23.9508	25.4867	25.7873	27.8713	**28.4915**	27.3360
15	24.0929	24.4846	26.5509	26.7164	30.4343	**30.7897**	29.9927
16	23.5826	24.1387	26.3258	27.0410	30.6613	30.9932	**31.0541**
17	23.8864	24.3835	26.5670	27.2550	30.8645	31.2523	**31.3186**
18	23.5707	24.0102	25.1366	25.9099	27.5905	28.0707	**28.0735**
19	23.5820	24.0696	26.0365	26.6651	29.8626	**30.3696**	30.2485
20	24.6759	25.0065	26.8145	27.1831	27.4626	28.4592	**30.0495**
21	23.5265	24.0586	25.6518	26.4513	28.9960	29.4035	**29.6244**
22	23.4976	24.0450	25.7935	26.3496	29.1417	**29.5544**	29.3926
23	23.6690	24.3757	26.2651	26.2849	32.3738	**32.5457**	30.7539
24	23.4244	23.8393	24.8499	25.8906	27.4968	28.0037	**28.1189**
Avg.	23.6711	24.1279	25.8796	26.4436	29.4139	**29.8748**	29.6666

**Table 3:** Comparison of the FSIM values of the Kodak RGB images interpolated by the Bayer CFA, Sony RGBW CFA, denoised Bayer CFA, and proposed RGBW CFA with 20 dB noise levels.

Data	Bayer DLMMSE [7]	Bayer RI [11]	Sony RGBW [22]	Proposed	Akiyama [28]	BM3D-CFA [29]	Proposed + BM3D
1	0.8672	0.8781	0.8914	0.9244	0.9233	0.9292	**0.9524**
2	0.7978	0.8044	0.8313	0.8701	0.8878	0.9156	**0.9382**
3	0.7502	0.7671	0.7955	0.8574	0.9330	0.9391	**0.9516**
4	0.7961	0.8107	0.8373	0.8856	0.9223	0.9339	**0.9519**
5	0.9067	0.9152	0.9190	0.9418	0.9297	0.9337	**0.9595**
6	0.8688	0.8789	0.8857	0.9234	0.9189	0.9264	**0.9512**
7	0.8290	0.8404	0.8593	0.9052	0.9441	0.9466	**0.9631**
8	0.8991	0.9078	0.9171	0.9409	0.9462	0.9500	**0.9687**
9	0.7640	0.7807	0.8045	0.8644	0.9425	0.9397	**0.9549**
10	0.8024	0.8176	0.8357	0.8900	0.9302	0.9306	**0.9530**
11	0.8406	0.8512	0.8678	0.9103	0.9206	0.9310	**0.9504**
12	0.7999	0.8140	0.8309	0.8832	0.9124	0.9285	**0.9474**
13	0.9100	0.9177	0.9178	0.9431	0.9135	0.9250	**0.9499**
14	0.8684	0.8783	0.8913	0.9241	0.9170	0.9252	**0.9500**
15	0.7889	0.7998	0.8254	0.8738	0.9293	0.9383	**0.9539**
16	0.7934	0.8084	0.8290	0.8830	0.9123	0.9201	**0.9419**
17	0.8368	0.8505	0.8689	0.9127	0.9308	0.9338	**0.9564**
18	0.8802	0.8890	0.8985	0.9261	0.9028	0.9127	**0.9430**
19	0.8295	0.8410	0.8610	0.9051	0.9201	0.9312	**0.9512**
20	0.7783	0.7891	0.8195	0.8691	0.9440	0.9472	**0.9589**
21	0.8639	0.8735	0.8824	0.9205	0.9241	0.9305	**0.9541**
22	0.8263	0.8392	0.8578	0.9011	0.9029	0.9191	**0.9424**
23	0.7393	0.7570	0.7808	0.8504	0.9522	0.9432	**0.9554**
24	0.8417	0.8533	0.8687	0.9087	0.9234	0.9299	**0.9538**
Avg.	0.8283	0.8574	0.8401	0.9006	0.9243	0.9317	**0.9522**

**Table 4:** Comparison of the FSIM values of the Kodak RGB images interpolated by the Bayer CFA, Sony RGBW CFA, denoised Bayer CFA, and proposed RGBW CFA with 23dB noise levels.

Data	Bayer DLMMSE [7]	Bayer RI [11]	Sony RGBW [22]	Proposed	Akiyama [28]	BM3D-CFA [29]	Proposed + BM3D
1	0.9154	0.9221	0.9314	0.9497	0.9480	0.9513	**0.9668**
2	0.8657	0.8689	0.8924	0.9179	0.9146	0.9378	**0.9535**
3	0.8313	0.8436	0.8705	0.9079	0.9483	0.9555	**0.9642**
4	0.8650	0.8757	0.8986	0.9275	0.9426	0.9525	**0.9653**
5	0.9413	0.9465	0.9478	0.9611	0.9558	0.9579	**0.9733**
6	0.9156	0.9215	0.9304	0.9489	0.9468	0.9503	**0.9656**
7	0.8878	0.8957	0.9132	0.9386	0.9612	0.9644	**0.9738**
8	0.9345	0.9401	0.9465	0.9609	0.9635	0.9668	**0.9779**
9	0.8398	0.8522	0.8747	0.9128	0.9599	0.9595	**0.9671**
10	0.8715	0.8815	0.9007	0.9313	0.9523	0.9528	**0.9681**
11	0.8950	0.9023	0.9161	0.9407	0.9433	0.9505	**0.9653**
12	0.8689	0.8785	0.8939	0.9250	0.9345	0.9466	**0.9602**
13	0.9428	0.9471	0.9456	0.9596	0.9453	0.9500	**0.9661**
14	0.9148	0.9211	0.9307	0.9501	0.9430	0.9477	**0.9645**
15	0.8565	0.8641	0.8875	0.9176	0.9480	0.9545	**0.9650**
16	0.8634	0.8735	0.8922	0.9257	0.9374	0.9428	**0.9591**
17	0.8936	0.9020	0.9186	0.9440	0.9518	0.9529	**0.9694**
18	0.9225	0.9285	0.9354	0.9502	0.9397	0.9439	**0.9615**
19	0.8888	0.8971	0.9136	0.9386	0.9423	0.9491	**0.9648**
20	0.8510	0.8576	0.8882	0.9166	0.9586	0.9627	**0.9695**
21	0.9131	0.9194	0.9271	0.9484	0.9489	0.9521	**0.9685**
22	0.8875	0.8962	0.9124	0.9353	0.9290	0.9409	**0.9579**
23	0.8241	0.8375	0.8606	0.9050	0.9657	0.9611	**0.9665**
24	0.8982	0.9059	0.9180	0.9403	0.9473	0.9516	**0.9673**
Avg.	0.8870	0.8949	0.9102	0.9356	0.9470	0.9523	**0.9659**
